# 'Prechronous' metastasis in clear cell renal cell carcinoma: a case report

**DOI:** 10.1186/1752-1947-5-181

**Published:** 2011-05-13

**Authors:** Eileen Poon, Sin Jen Ong, Xue En Chuang, Wan Teck Lim, Nor Azhari Mohd Zam, Tsung Wen Chong, Issam Al Jajeh, Kent Mancer, Min-Han Tan

**Affiliations:** 1 Department of Medical Oncology, National Cancer Centre Singapore; 2 Department of Urology, Singapore General Hospital, Singapore; 3 Department of Pathology, Singapore General Hospital, Singapore; 4 Department of Pathology, Changi General Hospital, Singapore; 5 NCCS-VARI Laboratory of Translational Cancer Research, National Cancer Centre, Singapore

## Abstract

**Introduction:**

Although metastatic carcinoma in the presence of an occult primary tumor is well recognized, underlying reasons for the failure of the primary tumor to manifest are uncertain. Explanations for this phenomenon have ranged from spontaneous regression of the primary tumor to early metastasis of the primary tumor before manifestation of a less aggressive primary tumor. We report a case of 'prechronous' metastasis arising from clear cell renal cell carcinoma, where metastatic disease initially manifested in the absence of a primary renal tumor, followed by aggressive growth of the primary renal lesion.

**Case presentation:**

A 43-year-old Malay man initially presented to our facility with fever and cough. He subsequently underwent surgical resection of a 9 cm right-sided lung mass found on radiological examination. Histology showed a high-grade clear cell tumor with sarcomatoid differentiation, suggestive of a metastasis from clear cell renal cell carcinoma. However, no concurrent renal lesions were noted on computed tomographic evaluation at that time. Then, four months after lung resection, he presented with a subcutaneous mass in the left loin, as well as right loin discomfort. Computed tomography scanning revealed a 10 cm right renal mass, with renal vein and inferior vena cava invasion, as well as recurrent disease in the right thorax. Histological examination of the excised subcutaneous mass revealed a high-grade carcinoma consistent with clear cell renal cell carcinoma.

**Conclusions:**

This is the first reported case of prechronous metastasis of renal cell carcinoma, with metastatic disease manifesting prior to the development of the primary lesion. The underlying mechanism is uncertain, but our patient's case provides anecdotal support for the early dissemination model of metastasis.

## Introduction

Although metastatic carcinoma in the presence of an occult primary is well recognized as a common clinical scenario of 'carcinoma of unknown primary' [1], underlying reasons for the failure of a primary tumor to manifest are uncertain. Possible explanations have ranged from spontaneous regression of the primary to an early metastasis. We report a case of 'prechronous' metastasis (see Discussion) arising from clear cell renal cell carcinoma (RCC), with the primary lesion manifesting only after the metastatic lesion was resected.

## Case presentation

**Figure 1:**
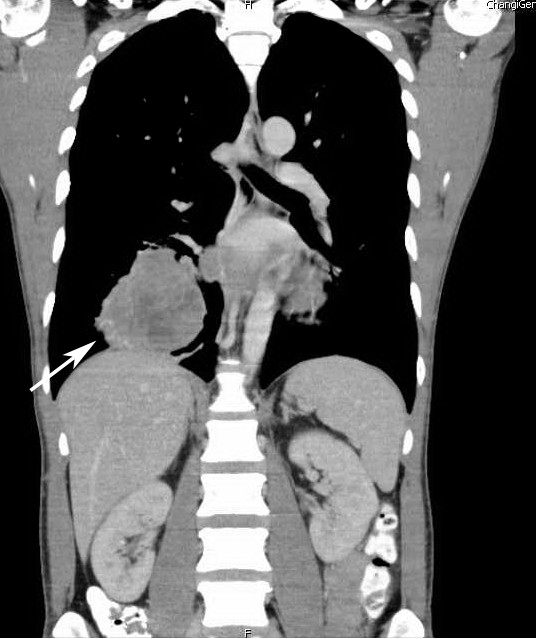
**Computed tomography (CT) coronal view of our patient's thorax and abdomen, showing a large right lower lobe lesion (arrow)**. As shown here, the kidneys were free of any lesions.

A 43-year-old Malay man presented to our facility with a three-month history of fever, non-productive cough and weight loss. He was a chronic smoker and had no significant medical history. Results of a physical examination were unremarkable. A chest radiograph revealed a large right lower zone lung lesion, and a subsequent computed tomography (CT) scan of the thorax and abdomen revealed a large heterogeneously enhancing soft tissue mass in the right lower lobe of the lung with intra-cavitary extension into the left atrium via the right inferior pulmonary vein (Figure 1). Transthoracic needle aspiration of this mass was suggestive of carcinoma. Surgery was performed for the resection of this mass; a right posterior lateral thoracotomy was performed, followed by a right lower lobectomy. The left atrium was opened at the inferior part of the superior pulmonary vein and the tumor resected with a small cuff of left atrium. The entire tumor and right lower lobe was delivered en bloc, and the left atrial defect subsequently patched. Histology demonstrated a high-grade clear cell sarcomatoid tumor, suggestive of metastatic clear cell renal cell carcinoma, a diagnosis specifically considered by the pathologist. On immunohistochemistry, the lesion was focally positive for epithelial membrane antigen (EMA), CD10 and vimentin, but negative for anticytokeratin CAM5.2, thyroid transcription factor-1 (TTF-1), smooth muscle actin (SMA), S100, HMB-45, Melan-A, Hepar and synaptophysin. However, as no renal lesion was evident on the CT scan (Figure 1), a diagnosis of alveolar soft part sarcoma was considered. An additional extensive investigation did not reveal a primary lesion or any other metastatic lesions.

**Figure 2:**
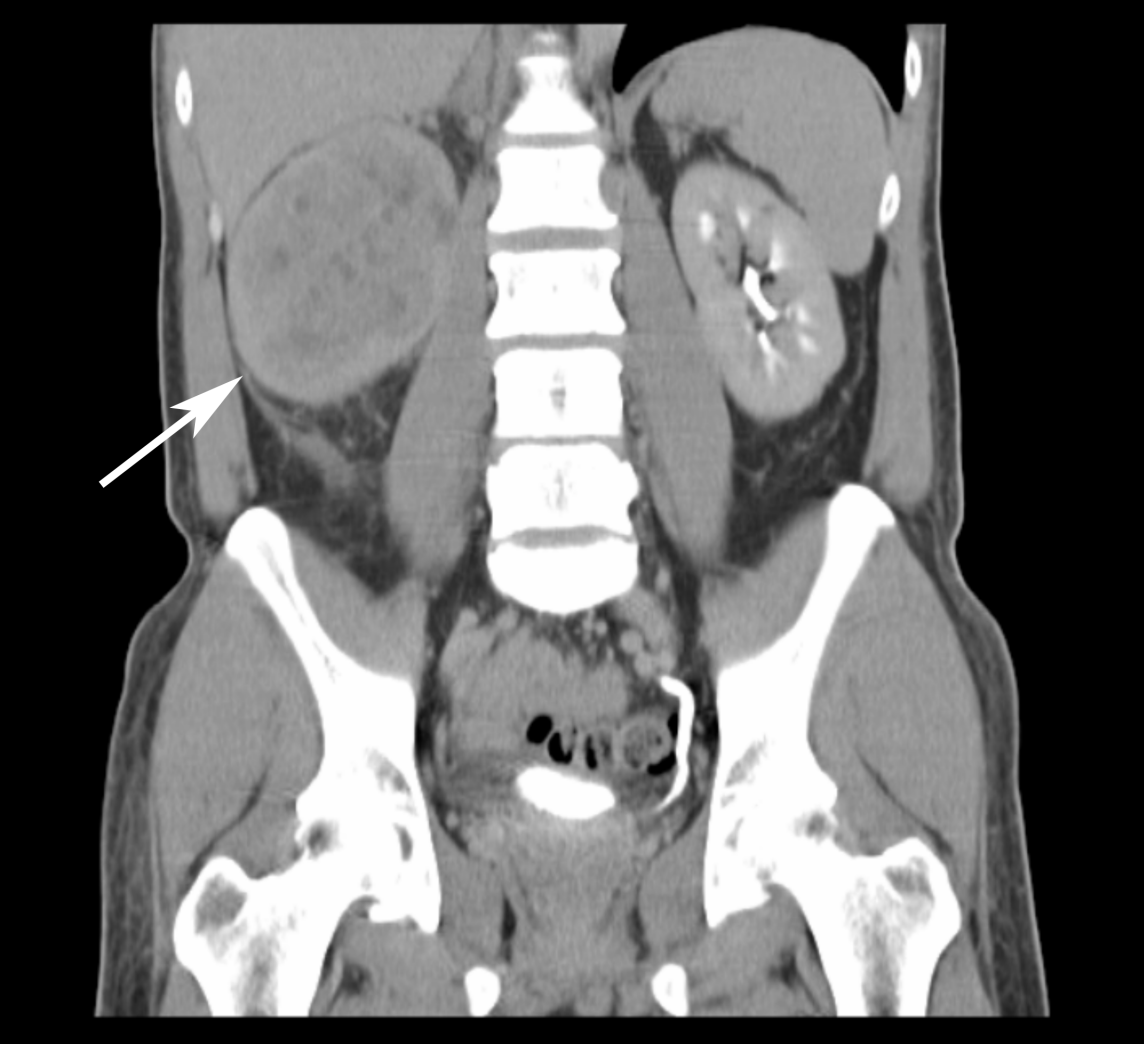
**Computed tomography (CT) coronal view of our patient's thorax and abdomen, showing a large right renal cell carcinoma (arrow) 4 months later**. This image is in the same coronal cut as Figure 1, as can be seen from evaluation of the vertebral column.

**Figure 3:**
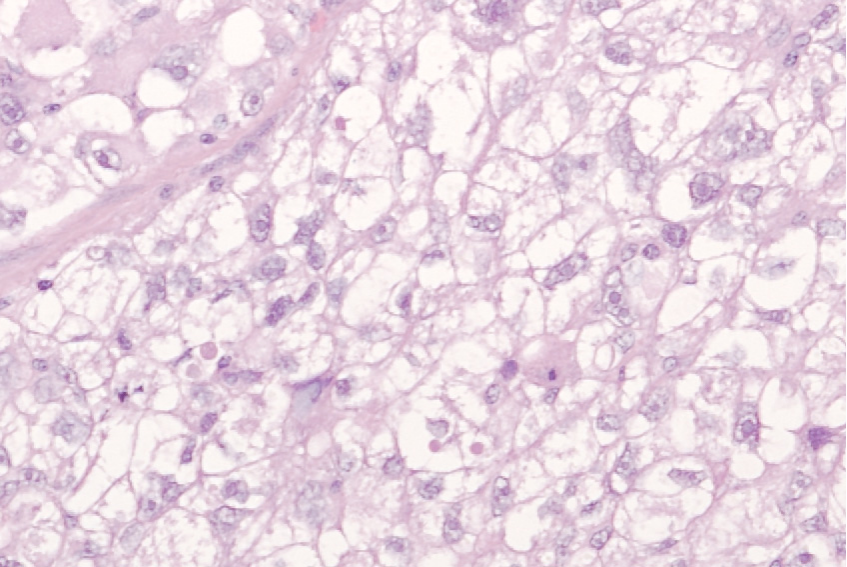
**Histology of the lung tumor showing a clear cell malignancy at (a) 20 × magnification and (b) 40 × magnification**.

**Figure 4:**
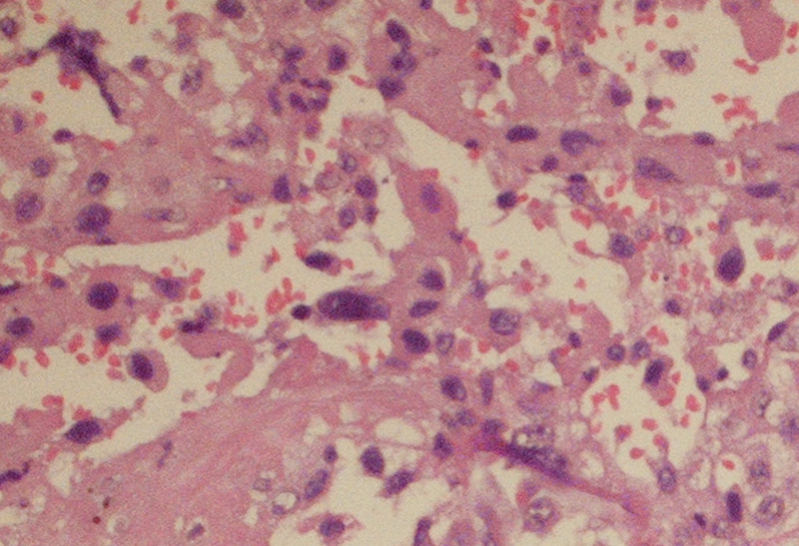
**(a) Hematoxylin and eosin staining of the resected subcutaneous nodule; (b) immunostaining for CD10, (c) epithelial membrane antigen. and (d) vimentin**. Magnification is 20 × for all images.

Then, four months later, our patient developed a subcutaneous mass in his left loin. A CT scan of the abdomen confirmed a large 11 cm tumor occupying nearly the entire right kidney with involvement of the pelvicalyceal system and proximal ureter (Figure 2). The tumor also extended into the right renal vein and the inferior vena cava, with a 2 cm soft tissue nodule was seen in the subcutaneous layer of the left flank. Further imaging of the thorax demonstrated multiple lung nodules, a large right pleural-based mass and an enlarged subcarinal lymph node. A bone scan was performed, and suggested involvement of the right humeral head and multiple thoracic vertebrae. Excision biopsy of the subcutaneous nodule was performed, and histology demonstrated a tumor morphologically similar to the initially resected lung lesion, suggestive of a high-grade clear cell renal cell carcinoma with sarcomatoid differentiation (Figure 3). On immunohistochemistry, the tumor was strongly positive for vimentin, CD10, focally positive for epithelial membrane antigen, melan-A and negative for TTF-1, S100, inhibin and synaptophysin (Figure 4) The positive vimentin and negative inhibin results weighed against the likelihood of an adrenocortical tumor.

Our patient was given palliative first-line therapy of sunitinib, with initial best response of stable disease. After three cycles of sunitinib, the disease progressed; our patient declined any further therapy and he eventually died 13 months after his initial lung resection.

## Discussion

About 25% to 30% of patients with RCC present with metastatic disease at diagnosis but less than 5% have solitary metastasis. Tumors with sarcomatoid change often have poorer prognosis. Our patient presented initially with a symptomatic metastasis in the absence of an evident primary; the primary tumor manifested only subsequently following metastatectomy. This phenomenon has been reported once before in the setting of lung cancer, where a 51-year-old woman presented with symptomatic brain metastasis [2], where the lung primary was eventually detected in the left upper lobe five years after resection. We sought a term to best describe this phenomenon. The terms 'synchronous metastasis' and 'metachronous metastasis' are well understood in terms of timing relative to the development of the primary tumor. The former term refers to a concurrent manifestation of metastasis and primary tumor, whereas 'metachronous' refers to the subsequent development of metastasis. Using a similar Greek prefix, the term 'prechronous' clearly describes the phenomenon observed here, where a metastatic lesion manifests prior to the primary lesion. Ours represents the first such report of this phenomenon in renal cell carcinoma, and we briefly discuss possible hypotheses here that may underpin this.

In the standard late dissemination model of metastasis, the metastatic cascade [3] is a multi-step sequential process in which cancer cells depart from the primary tumor and enter the lymphatics, blood or body cavity. They deposit at nearby or distant sites before proliferating to colonize ectopic tissues. It is recognized that metastases have a predilection for certain sites [4] and require that these key sites be first seeded [3]. However, there has been recent evidence to support aspects of the early dissemination model, where metastasis occurs early in the life cycle of carcinogenesis. Podsypanina *et al*. engineered untransformed mouse mammary cells to express inducible oncogenes transgenes that are able to bypass the primary site and phenotypically show up at secondary sites [5]. Kaplan *et al*. also showed that cancer cells in murine models may relay signals, involving vascular endothelial growth factor receptor 1 (VEGFR1) and fibronectin, to bone marrow cells to migrate to distant organs to establish an environment amenable to metastasis [6]. This phenomenon preceded the formation of micrometastatic colonies in these organs by four to six days. Our case report provides anecdotal but direct support for the early dissemination model of metastasis.

There are some clinical similarities between our case report as described, and the phenomenon of 'burned-out' cancers seen most commonly in germ cell tumors. In the clinical setting of patients with 'burned-out' germ cell tumors, metastatic lesions are first identified in the presence of regressed primary tumors, the latter diagnosed by a distinct histological appearance [7,8]. However, our case report differs in demonstrating a clear aggressive behavior for the primary tumor upon clinical manifestation post-metastatectomy, with radiological growth from undetectable to an 11 cm lesion over four months, which is inconsistent with a 'burned-out' primary.

## Conclusions

We report a case of sarcomatoid clear cell RCC, demonstrating the rare phenomenon of prechronous metastasis. Our report provides direct support for the early dissemination model of metastasis.

## Consent

Written informed consent was obtained from the patient for publication of this case report and any accompanying images. A copy of the written consent is available for review by the Editor-in-Chief of this journal.

## Competing interests

The authors declare that they have no competing interests.

## Authors' contributions

NAMZ, WTL, CTW and TMH were involved in the clinical care of our patient; IAJ and KM performed the histological examinations. EP, OSJ, CXE and TMH were major contributors to the manuscript writing. All authors read and approved the final manuscript.
